# Surface energy and stress driven growth of extremely long and high-density ZnO nanowires using a thermal step-oxidation process†

**DOI:** 10.1039/d4ra03128h

**Published:** 2024-09-03

**Authors:** Sri Aurobindo Panda, Sumita Choudhary, Sushil Barala, Arnab Hazra, Suchit Kumar Jena, Subhashis Gangopadhyay

**Affiliations:** a Department of Physics, Birla Institute of Technology & Science Pilani Rajasthan India; b Department of Electrical and Electronics Engineering, Birla Institute of Technology & Science Pilani Rajasthan India subha@pilani.bits-pilani.ac.in; c Department of Physics, Indian Institute of Technology Guwahati Guwahati 781039 Assam India

## Abstract

Formation of highly crystalline zinc oxide (ZnO) nanowires with an extremely high aspect ratio (length = 60 μm, diameter = 50 nm) is routinely achieved by introducing an intermediate step-oxidation method during the thermal oxidation process of thin zinc (Zn) films. High-purity Zn was deposited onto clean glass substrates at room temperature using a vacuum-assisted thermal evaporation technique. Afterwards, the as-deposited Zn layers were thermally oxidized under a closed air ambient condition at different temperatures and durations. Structural, morphological, chemical, optical and electrical properties of these oxide layers were investigated using various surface characterization techniques such as X-ray diffraction (XRD), scanning electron microscopy (SEM), Raman spectroscopy, and X-ray photoemission spectroscopy (XPS). It was noticed that the initial thermal oxidation of Zn films usually starts above 400 °C. Homogeneous and lateral growth of the ZnO layer is usually preferred for oxidation at a lower temperature below 500 °C. One-dimensional (1D) asymmetric growth of ZnO started to dominate thermal oxidation above 600 °C. Highly dense 1D ZnO nanowires were specifically observed after prolonged oxidation at 600 °C for 5 hours, followed by short-step oxidation at 700 °C for 30 minutes. However, direct oxidation of Zn films at 700 °C resulted in ZnO nanorod formation. The formation of ZnO nanowires using step-oxidation is explained in terms of surface free energy and compressive stress-driven Zn adatom kinetics through the grain boundaries of laterally grown ZnO seed layers. This simple thermal oxidation process using intermittent step-oxidation was found to be quite unique and very much useful to routinely grow an array of high-density ZnO nanowires. Moreover, these ZnO nanowires showed very high sensitivity and selectivity towards formaldehyde vapour sensing against few other VOCs.

## Abstract 2

Formation of ZnO nanowires during thermal oxidation of thin Zn films.

## Graphical abstract

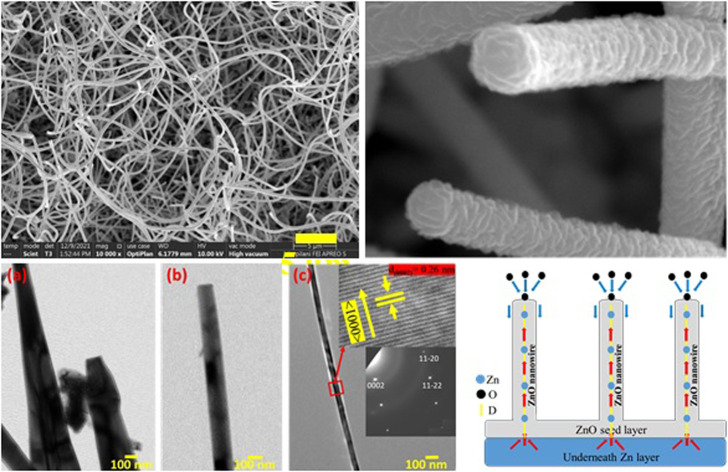

## 1 Introduction

Nanoscale surface geometry of any semiconducting material can play a very crucial role in its device performance. Hence, to exploit its maximum device potential, the ability to control its surface morphology down to the nanometer length scale is of high technological demand. Among the various low-dimensional semiconducting materials, one-dimensional (1D) nanostructures such as nanowires or nanorods exhibit unique properties as they provide a structurally asymmetric platform to study the impact of nanoscale size on their performance. Here, this one dimension (length) is dramatically larger and may adjust independently with respect to the other two dimensions (diameter). Moreover, these 1D nanostructures may significantly alter their physical, chemical and electronic properties by varying the size, shape, phase, stoichiometry, facets and defects. Owing to these extraordinary properties, 1D nanostructures have attracted the attention of a large number of material scientists.^1,2^ However, the controlled formation of complex one-dimensional nanostructures with a well-defined lattice orientation and surface morphology is still a great challenge. A basic understanding of this unidirectional growth mechanism can successfully address the controlled formation of 1D nanostructures and their various functionalities.

Spontaneous formation of 1D metal oxide-based nanostructures such as nanowires/whiskers together with their underlying growth mechanism has been a controversial topic for many years. Some of the established mechanisms of 1D growth during deposition are based on vapour–liquid–solid (VLS) and vapour–solid (VS) models. The VLS method is proposed with an extra droplet (metal surfactant) at the tip of the 1D nanostructure, which catalyzed the absorption and condensation of the material vapour to sustain the directional asymmetric growth. However, in the case of VS mechanism, direct deposition of the gaseous components onto substrates occurs to form 1D nanostructures. Another possibility for 1D nanostructure growth during the deposition process could be the self-catalytic growth.

Apart from the direct deposition of metal oxide layers, other approaches for growing metal oxide nanowires include the thermal oxidation of thin metal films where oxide nanowires can be formed due to the accumulation and relaxation of compressive stresses.^3–6^ In general, there are two possible migration pathways for the metal ions during the thermal oxidation process. One of them is the internal diffusion along a tunnel centered on the core of a screw dislocation or a bicrystal grain boundary,^4,6^ whereas the other one is the surface diffusion along the side walls of the nanowires.^3,5,7^ Due to the transportation of cations of metal oxides from the base layer to the tip of the structure, metal oxide nanowires continue to grow from the nib and not from the base layers of the oxide. Hence, direct oxidation of metals may lead to an asymmetric growth of metal oxides if the synergetic effects of oxidation temperature and duration are properly controlled, where the diffusion of metal ions from the nanowire base to the tip either along the outer surface or along a well-centered core of a crystallographic defect results in 1D nanowire growth. These mechanisms of NW formation for CuO, Fe_3_O_4_ or Co_3_O_4_ are discussed in detail where a double-oxide junction layer such as CuO/Cu_2_O, Fe_2_O_3_/Fe_3_O_4_ or CoO/Co_3_O_4_ is formed as the base layer. We can also extend this idea for the growth of ZnO nanowires during the thermal oxidation process. At a higher temperature, 1D nanorods of ZnO are usually formed with a prismatic or pyramidal type morphology. The dominance of two non-polar planes such as (10–10) and (10–11) over (0002) is the key factor to produce the highly crystalline and dense ZnO nanorods.^2^ Here, the surface morphology of the oxide material largely depends on the crystalline orientation and growth kinetics of the crystal growth front. The surface free energy of those crystalline facets is equally important to determine the final growth morphology. Direct evidence of CuO NW formation using unidirectional Cu ion outer diffusion through a bicrystal grain boundary has already been reported.^6^ Outer surface diffusion of Cu ions for NW growth has also been reported.^3^ However, there is significant lack of evidence explaining the ZnO nanowire growth mechanism. Therefore, a detailed analysis of the thermally grown ZnO nanowire would be of high scientific importance.

In general, ZnO-based nanostructures are very attractive as they can appear in various morphologies starting from three-dimensional (3D) bulk, two-dimensional (2D) quantum well, one-dimensional (1D) quantum wires to zero-dimensional (0D) quantum dots. Among all these, 1D nanowires have attracted considerable attention due to their high surface area-to-volume ratio along with highly flexible geometry. As they provide a novel platform to explore the excellent nanoscale phenomena, a reliable, simple and cost-effective growth method is highly necessary to fully explore these unique properties for various electronic and other applications. Among all the growth methods of ZnO, thermal oxidation is found to be the simplest, fast and efficient approach to grow the oxide nanostructures. Various oxidation parameters such as annealing/oxidation temperature and duration, oxygen partial pressure of the ambience, and metal film thickness can systematically be explored here. Although, the classical Mott model has successfully explained the oxidation behaviour of metal films assuming a continuous and uniform oxide growth, the formation mechanism behind the anisotropic growth of 1D ZnO nanowires has not yet been well understood. Therefore, a detailed understanding of its controlled morphological evolution towards nanowire formation along with the thermal diffusion and mass transport mechanism is very much useful.

In recent years, ZnO-based nanomaterials have gained vast scientific interest because of their exciting physical and electronic properties. In general, ZnO shows a wurtzite crystal structure and possesses a direct bandgap of 3.37 eV. Intrinsic ZnO usually behaves like an n-type semiconductor due to its unintentional oxygen vacancies, which can significantly alter its surface electronic and optical properties. ZnO has also received the attention of the scientific community due to its unique piezoelectric property^8^ and large exciton binding energy (60 meV).^9^ Its room-temperature exciton recombination property can be used in laser diodes. Due to the electron confinement effect, the transport and optical properties of ZnO nanowires have largely been enhanced. All these interesting properties make ZnO-based nanomaterials very useful for various applications in the field of sensors, optoelectronics, photocatalysts, piezoelectric transducers and actuators.^10–14^

It has already been reported that ZnO-based nanomaterials may appear in a wide variety of 1D nanostructures such as nanowires,^15–17^ nanorods,^18–20^ nanotubes,^21–23^ nanobelts,^24–26^ and nanoneedles.^27^ In addition to the various structures, excellent surface redox chemistry and tuneable surface electrical conductance of ZnO make it one of the most useful materials in the field of gas sensor applications to detect various ultra-sensitive VOCs, toxic gases and DNA.^28–33^ In addition, enhanced thermal and chemical stability of ZnO makes it a useful material in the ceramic industry. The transparent phase of zinc oxide nanowires at intermediate oxidation temperatures makes it also useful for the solar cell application.^34–37^

Moreover, 1D ZnO nanostructures may serve as templates for further growth of hybrid nanostructures. Recently, electron beam-induced formation of hybrid nanostructures of ZnO nanocrystals on ZnO nanoneedles has been reported, which may hold great promise for enhanced photovoltaic performance.^38^ Another ZnO-based hybrid nanostructure of tetrapod for nanowire formation using Au and Sn-assisted vapour phase transport (VPT) has also been reported.^39,40^ These hybrid nanostructures show an excellent luminescence property in the UV region, and hence, they can be promising candidates for optoelectronic applications. However, in-assisted VPT of ZnO may lead to the formation of large 2D layered structures.^41,42^

A comparative analysis of various reported ZnO nanowires, grown *via* different synthesis routes, is summarized in Table 1. Mostly, ZnO nanowires are grown using various chemical growth routes, among which the hydrothermal method is widely used.^16,43–46^ In addition, solution^47^ and sol–gel^48^ methods are explored. Apart from chemical routes, reactive sputtering and CVD methods^49,50^ are reported. Thermal oxidation of sputter deposited metal films and thin foils is also reported.^51–53^ In general, chemical growth methods usually result in the formation of ZnO nanowires with a shorter length and a wider diameter having an aspect ratio of ∼20–40. In contrast, thermal oxidation of Zn films provides a higher aspect ratio of ∼100 for the ZnO nanowires. In addition, the physical growth route provides much higher physical and thermal stability for the as-grown ZnO nanowires along with high material purity. Hence, the physical growth method of ZnO will be more preferable, where material stability and purity are crucial.

**Table 1:** Comparative study of ZnO nanowires grown using various synthesis methods

Growth method	Length (μm)	Diameter (nm)	Aspect ratio	Ref.
Hydrothermal	4.8	160	30	16
Hydrothermal	0.375	60	6.3	43
Hydrothermal	0.845	27.2	31.1	44
Hydrothermal	1	50	20	45
Hydrothermal	2.8	78	35.9	46
Solution process	25	200	125	47
Sol–gel template	3.6	70	51.4	48
Vapor trapping CVD	30	100	300	49
CVD	1	30	33.3	50
Sputtering (O_2_/Ar)	2.7	45	60	51
Sputtered thermal oxidation (Ar)	1.1	15	73.3	52
Thermal oxidation (air)	50	75	667	53

Among all the above-mentioned ZnO nanowire growth methods, the thermal oxidation of thin Zn films is a simple and straight forward process that does not require any complex and expensive equipment, which makes it an attractive method for producing ZnO nanowires at a relatively low cost. Moreover, this process can be easily scaled up for large-scale production for industrial applications. During this growth process, oxidation parameters such as temperature, time, and ambient atmosphere can precisely be controlled, which allows the fine-tuning of the size, morphology, and density of ZnO nanowires, for optimizing their properties for specific applications. Moreover, the thermal oxidation process typically results in high purity and good crystalline quality of ZnO nanowires, which are very crucial for any device and sensing applications. As Zn thin films can easily be deposited using any standard evaporation technique commonly used in the semiconductor industry, subsequent thermal oxidation to produce ZnO nanowires may also be integrated into the existing manufacturing processes. Finally, the thermal oxidation process is an environmental friendly process as it generally does not require any toxic chemicals or produce hazardous by-products, an emphasis on green manufacturing processes. Therefore, the thermal oxidation of Zn films to grow extremely long nanowires would be of high technological importance.

A large number of scientific works have been reported on the growth of ZnO nanowires using different methodologies (Table 1); however, very few of the methodologies result in the formation of long nanowires (above of 10 μm), particularly *via* a thermal oxidation process (48). A new approach of intermittent step-oxidation (prolonged thermal oxidation at 600 °C for 5 h followed by short-duration step-oxidation at 700 °C for 30 min) was introduced here to routinely achieve extremely long ZnO nanowires of high density. Surprisingly, direct oxidation at 700 °C resulted in the formation of ZnO nanorods of much shorter length. To the best of our knowledge, this intermittent step-oxidation is a completely new methodology of thermal oxidation process, which can successfully lead to the formation of ZnO nanowires as long as 60 μm. In order to check the reproducibility of this method, different samples were prepared following the same thermal treatment over the months and a very similar type of surface morphology was observed. We would like to emphasize that the growth of extremely long ZnO nanowires of high crystalline quality has rarely been reported, and due to their high density and enhanced active surface area, these nanowires are expected to be highly efficient in gas sensing and photovoltaic applications. Overall, this step-oxidation method offers a practical and efficient route for ZnO nanowire growth, which can further be integrated into any existing and emerging technologies.

In this work, we present a unique and simple growth mechanism of an array of ZnO nanowires along with their analytical studies using thermal evaporation of Zn films followed by a thermal oxidation process. A new approach of an intermittent step-oxidation process was exploited here for the growth of extremely long ZnO nanowires. In addition, compared to most of the reported ZnO nanowire growth methods, our air ambient step-oxidation method appears to be very simple and much superior in terms of the aspect ratio of the ZnO nanowires (∼1200) and their crystalline quality. The main purpose of this work was to validate a simple growth methodology, which can successfully grow highly crystalline and extremely long nanowires of ZnO and make this simple thermal oxidation process a very good alternative to various ZnO nanowire growth methods.

## 2 Experimental details

**Fig. 1:**
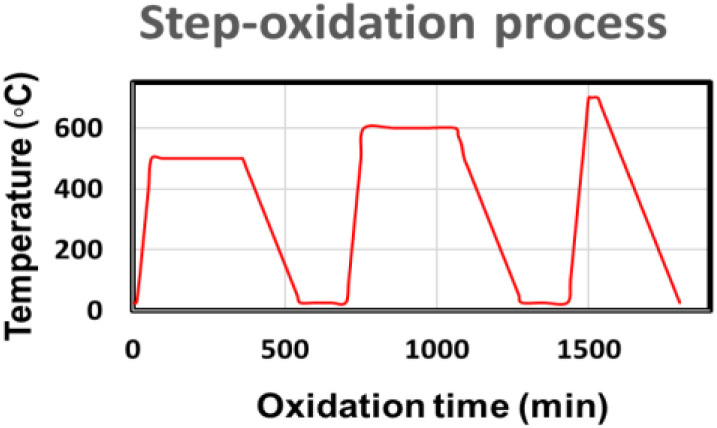
Schematic of the thermal step-oxidation process for the growth of extremely long ZnO nanowires.

All substrates (glass) were chemically cleaned with ethanol, methanol, and acetone solutions using an ultrasonicator and then properly dried before loading into the vacuum deposition chamber (HINDHIVAC). Afterwards, high-purity Zn was deposited onto a glass substrate at room temperature by a vacuum-assisted thermal evaporation method, where a molybdenum boat was used to evaporate the Zn metal. During the deposition process, the base pressure of the deposition chamber was maintained at ∼10^−5^ mbar. Subsequently, Zn films were vacuum annealed *in situ* using a substrate heater to enhance their adherence to the substrates. In order to form zinc oxide films, thermal oxidation of the as-deposited zinc films was performed stepwise at different oxidation temperatures ranging from 200 °C to 700 °C under an air ambient condition using a muffle furnace (TEMPCON). The oxidation duration was fixed at 5 hours for each oxidation step up to 600 °C and the samples were cooled down to room temperature after each step. Finally, short-duration annealing at 700 °C for 30 minutes was carried out to grow highly dense ZnO nanowires. For better clarity, a schematic representation of the thermal step-oxidation process is shown in Fig. 1. The structural, morphological, chemical, electronic and optical properties of the as-grown ZnO nanowires have been investigated precisely during each step-wise oxidation process. The crystalline quality of the as-grown ZnO films was studied using a Rigaku Miniflex II X-ray diffractometer (XRD), where copper K-alpha line was used as the X-ray source (*λ* = 0.154 nm). FESEM technique was performed to analyze the surface morphology of the as-grown ZnO films. The Raman spectra of the as-grown ZnO nanowires confirm the oxide phases. Finally, the oxidation state and chemical compositions of the as-grown ZnO nanowires were studied by XPS. For statistical analysis of the as-grown ZnO nanowires as well as validation of the step-oxidation process for extremely long ZnO nanowire formation, two different repeatability studies were adopted: (a) different samples prepared during the same oxidation process and (b) different samples prepared *via* different oxidation processes of similar thermal treatment. Finally, these ZnO nanowires were used to fabricate a chemi-resistive gas sensor, by depositing Au finger electrodes on top to investigate their sensing ability against different VOCs.

## 3 Results and discussion

### 3.1 Structural characterization by XRD

**Fig. 2:**
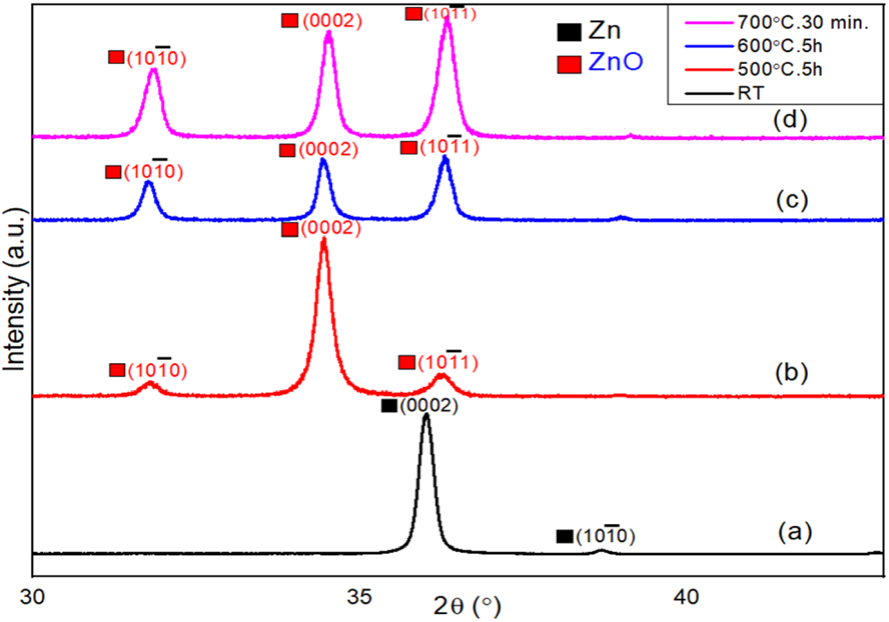
XRD spectra of (a) the as-deposited metallic Zn film at room temperature. Thermally oxidized Zn thin films at (b) 500 °C for 5 h, (c) 600 °C for 5 h, and (d) 700 °C for 30 min.

X-ray diffraction technique was used to investigate the crystalline quality and oxidation steps of Zn thin films during the thermal oxidation process. Fig. 2 shows the XRD patterns of Zn films thermally oxidized at different temperatures. Fig. 2(a) represents the XRD spectrum of the as-deposited metallic Zn thin films at room temperature. Strong diffraction peak of the (0002) crystal plane of metallic Zn appears at 2*θ* = 36°, confirming the hexagonal close packed structure of Zn metal. In addition, the Zn (10–10) diffraction plane appears at 2*θ* = 39°, but with a much weaker intensity. In order to find the initial oxidation stage of the Zn film, the oxidation temperature was increased stepwise. Surprisingly, no diffraction peaks of the oxide structure are observed below the oxidation temperature of 400 °C, indicating that the thermal oxidation process of Zn films starts only above 400 °C.^54^ The XRD patterns of ZnO films grown at 500 °C for 5 hours are shown in Fig. 2(b), where the XRD peaks of metallic Zn completely disappear. Instead, the (0002) crystal planes of ZnO appear at 2*θ* = 34.5° along with two non-polar crystal planes of ZnO such as (10–10) and (10–11) at 2*θ* = 31.8° and 36.3°, respectively. The ZnO (0002) diffraction peak clearly dominates over other crystal planes, suggesting a lateral growth of polar planes (shown later in the FESEM results). Fig. 2(c) shows the XRD spectrum of Zn films after oxidation at 600 °C for 5 hours, where a mixture of polar (0002) and non-polar (10–11) and (10–10) planes are clearly observed. These findings suggest an asymmetric growth of 1D oxide nanostructures on top of the symmetric 2D lateral ZnO layer. In Fig. 2(d), the XRD pattern of this ZnO surface after short duration of annealing at 700 °C for 30 min is presented. The ZnO diffraction peak of the (10–11) crystal planes appears at 2*θ* = 36.3°, and dominates over all other peaks. This finding also indicates an asymmetric 1D growth of ZnO nanowires, which is further discussed in the following section (FESEM). All XRD patterns strongly corroborate the wurtzite crystal structure of the as-grown ZnO films with a hexagonal lattice, having lattice constants *a* = 0.324 nm and *c* = 0.519 nm. In addition, the average crystallite size of the as-grown ZnO nanowires was calculated to be about 30 nm, using the following Debye–Scherrer formula:*D* = *Kλ*/*β* cos *θ*,where *D* signifies the crystallite size, while *K* stands for the Scherrer coefficient (0.9), *λ* represents the wavelength of X-ray source (0.154 nm), *β* indicates the full width at half maximum (0.276 in radians) and *θ* represents the peak position (2*θ* = 36.3° for the 10–10 diffraction peak).

### 3.2 Morphological studies by FESEM

**Fig. 3:**
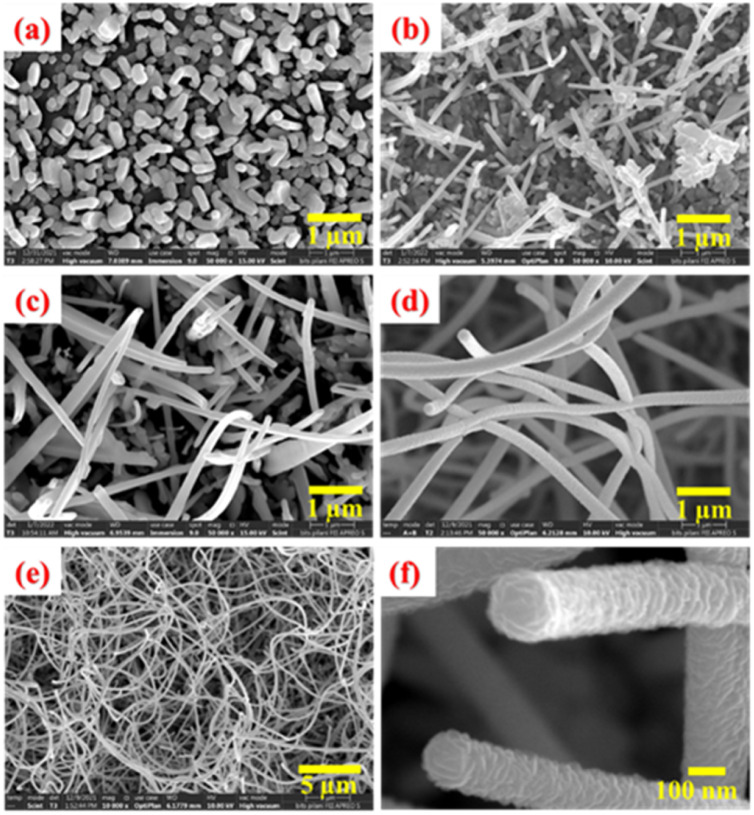
FESEM images of (a) as-deposited Zn thin films and as-grown ZnO films after oxidation at (b) 500 °C for 5 h, (c) 600 °C for 5 h and (d) 700 °C for 30 minutes. (e) Large scale and (f) closer view of samples shown in (d).

The morphological studies of the as-grown ZnO film surfaces were carried out using a field emission scanning electron microscope (FEI made). Fig. 3 represents the morphological evolutions of Zn surfaces during the thermal oxidation process at different temperatures and durations. Fig. 3(a) represents the surface morphology of the as-deposited Zn thin films. A closer view of the Zn surface depicts the hexagonal faceted surface morphology of the Zn nanoislands (not shown here). With the increase in oxidation temperature (above 400 °C), the oxidation process starts with a severe surface roughening. The surface morphology of the ZnO layer after oxidation at 500 °C for 5 hours duration is shown in Fig. 3(b). A homogeneously grown 2D lateral ZnO surface is preferred over few asymmetric 1D structures at this stage. Fig. 3(c) represents the asymmetric 1D growth of ZnO nanostructures after oxidation at 600 °C for 5 hours duration. This asymmetric 1D growth of ZnO is clearly dominated over the lateral 2D island growth. Finally, highly dense 1D nanowires completely cover the ZnO surface after further oxidation at 700 °C for a short duration of 30 minutes, as shown in Fig. 3(d).

For better statics, an overview FESEM image of these ZnO nanowires with a large-scale surface morphology is shown in Fig. 3(e). ZnO nanowires are uniformly grown throughout the ZnO surface with a very high special density. Moreover, these unidirectional ZnO nanowires are having an extremely high aspect ratio. To understand the growth steps more carefully, a closer look of the tips of two ZnO nanowires is depicted in Fig. 3(f). Spiral stacking of slate-like facet planes at the top of ZnO NWs are clearly observed. In addition, the side wall of these NWs are found to be quite rough, protruding the edges of spirally stacked slate-like structures. From all these findings, it can be assumed that these nanowires are grown along the *c*-axis of the ZnO crystal and the spiral stacking is around a screw dislocation at the core of the nanowire. It can also be related to the lower surface energy of the (10–11) and (10–10) crystal planes as compared to the (0002) *c*-planes of the ZnO crystal.^55–57^ In addition, a faster growth rate of the *c*-plane than that of the m-plane can also influence the anisotropic crystal growth of ZnO nanowires. Usually, these nanowires start to nucleate from the apex of the hexagonal ZnO nanosheets to minimizing the effect of spontaneous polarization and surface free energy of the (0002) plane. Lower surface energy of nonpolar crystal planes facilitates the asymmetric growth. Surprisingly, these dense nanowires are not observed for prolonged oxidation at 700 °C for 5 hours. Instead, it leads to the formation of ZnO nanorods of prismatic and pyramidal shapes.^58^ Therefore, it can be concluded that the intermittent step-oxidation process with limited supply of Zn adatoms is very much useful for the successful formation of ZnO nanowires. Apart from the surface free energy of crystal planes, Zn and O adatom kinetics and their stress-driven transport mechanism are crucial for 1D growth process, which are discussed later in the “Growth mechanism of ZnO nanowires” section.

In order to check the reproducibility of this growth method of extremely long ZnO nanowires, several samples were prepared following the same thermal treatment of prolonged step-oxidation at 600 °C for 5 hours duration followed by short-duration oxidation at 700 °C for 30 minutes. The heating rate was fixed to 10 °C min^−1^ and all samples were naturally cooled to avoid any thermal stress. Statistical analysis of these as-grown ZnO nanowires was performed by FESEM imaging and also to validate the step-oxidation growth methodology (Fig. SI 1†). Basically, two different growth approaches were adopted here. In one case, different samples were prepared during the same oxidation process such as Sample S1(a–c) and sample S2(a–b), whereas in other cases, the samples were prepared through different oxidation processes of similar thermal treatment S1–S4, within a time period of six months. The findings of different nanowire samples are summarized in Table SI 1.† The resulting SEM images are shown in Fig. SI 1,† which corroborate the occurrence of long ZnO nanowires. All seven samples appear with a very similar kind of ZnO nanowire surface morphology, with a minor deviation in their diameters and densities, which can be attributed to the temperature uncertainty with a range of 5 °C. The statistical mean value of the ZnO nanowires diameter was carefully calculated to be about 49 nm within a narrow range of deviation of ±2 nm. Finally, these findings for different nanowires can successfully validate the reliability of our step-oxidation process for ZnO nanowire growth.

### 3.3 TEM analysis of 1D ZnO nanostructures

**Fig. 4:**
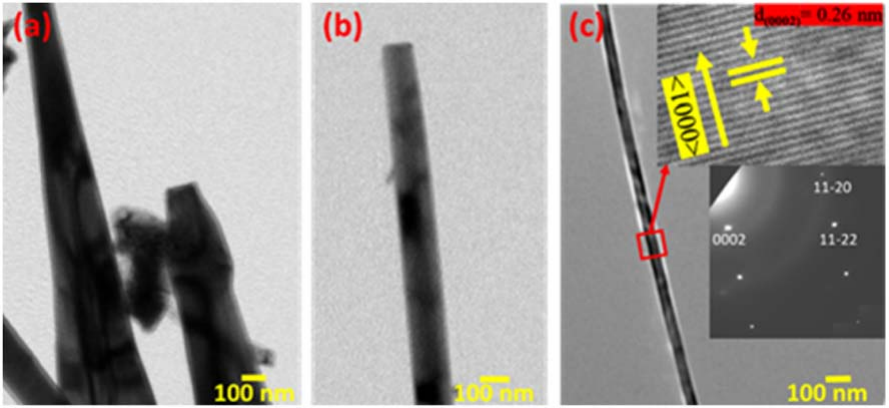
TEM image of different 1D ZnO nanostructures: (a) pyramidal nanorods, (b) prismatic nanorods and (c) nanowires. Inset (top): HRTEM image of the ZnO nanowire; inset (bottom): SAED pattern.

The TEM micrograph of different 1D ZnO nanostructures and the HRTEM image and SAED patterns of a ZnO nanowire are shown in Fig. 4. Fig. 4(a) shows the TEM micrograph of a pyramidal ZnO nanorod, whereas Fig. 4(b) represents a prismatic nanorod. A portion of an extremely long ZnO nanowire is depicted in Fig. 4(c). A gradual decrease in the 1D nanostructure diameter along with the increase in length is observed from Fig. 4(a) to (c). The HRTEM image of a selected area of ZnO nanowire and its corresponding SAED pattern are shown within the insets of Fig. 4(c). The inter-planer spacing was measured to be 0.26 nm, which corresponds to the lattice spacing of adjacent (0002) planes of wurtzite ZnO. The selected area diffraction pattern (SAED) pattern reveals that the nanowire is single crystalline in nature. Both HRTEM image and SAED pattern confirm the nanowire growth direction oriented along the *c*-axis of the ZnO crystal, *i.e.*, 〈0001〉 crystal direction of ZnO.^2^

### 3.4 Raman spectroscopy

**Fig. 5:**
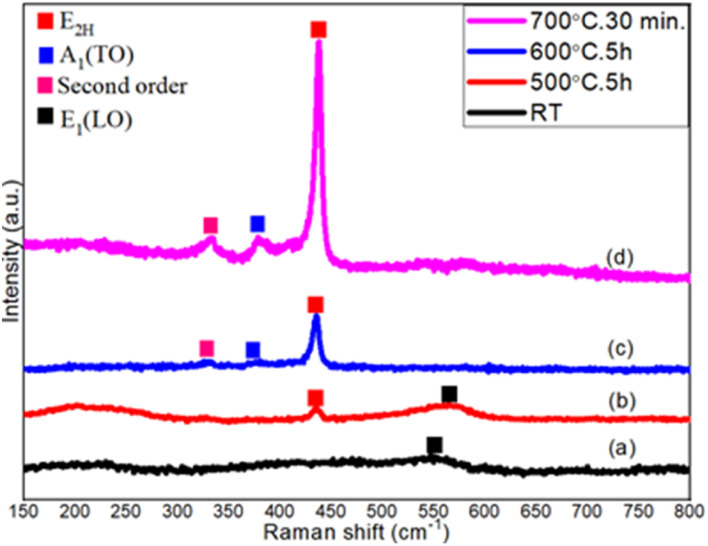
Raman spectra of (a) as-deposited metallic Zn thin films at RT, and thermally oxidized Zn films at (b) 500 °C for 5 h, (c) 600 °C for 5 h, and (d) 700 °C for 30 min.

Raman spectroscopy was performed to get the chemical composition of thermally oxidized ZnO thin films using a He–Ne laser source of wavelength *λ* = 633 nm. Fig. 5 describes the evolution of ZnO nanostructures starting from metallic Zn films, during the stepwise thermal oxidation process. In Fig. 5(a), E1(LO) mode appears at 553 cm^−1^ representing the metallic Zn phase. The E1(LO) mode indicates a severe oxygen deficiency within the metallic Zn or ZnO thin film. A clear shift of this mode towards a higher wave number at 567 cm^−1^ is observed after oxidation at 500 °C for 5 h (Fig. 5(b)). In addition, the E2H (known as the non-polar Raman-active optical phonon mode) also appeared at 436 cm^−1^ after oxidation at 500 °C, representing the wurtzite phase of ZnO. In Fig. 5(c), the E1(LO) mode finally disappears after oxidation at 600 °C for 5 hours and the intensity of the E2H mode gets significantly enhanced. The E2H mode also shows a blue-shift at 436.5 cm^−1^ (Fig. 5(c)). Two other modes of A1(TO) at 374 cm^−1^ and second order of E2H at 329 cm^−1^ are also observed at this oxidation temperature. After short step-oxidation at 700 °C for 30 min, the Raman-active E2H mode appears with a much stronger intensity and with a slight blue shift at 437 cm^−1^ (Fig. 5(d)). This indicates the highly crystalline nature of the strain-free ZnO nanowires. The polar A1 (TO) and second-order spectrum appear with a little blue-shift towards a higher wavenumber at 378 cm^−1^ and 333 cm^−1^, respectively. The blue shift with the increase in oxidation temperature can be related to the oxygen vacancy and different growth directions.

### 3.5 UV-visible spectroscopy

**Fig. 6:**
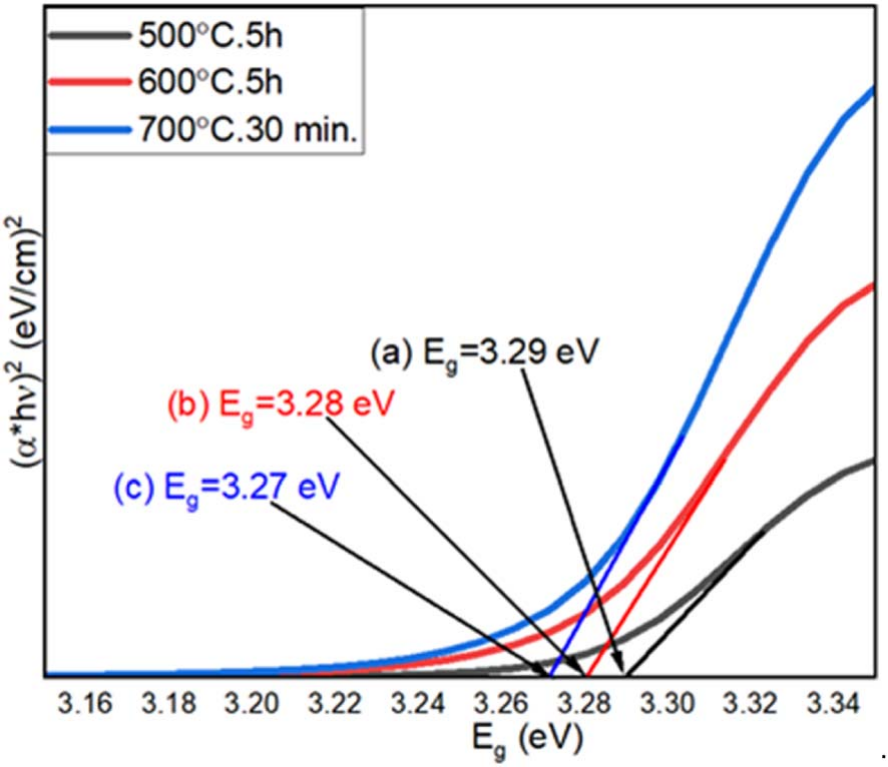
UV-visible spectra of thermally oxidized Zn thin films at (a) 500 °C for 5 h, (b) 600 °C for 5 h, and (c) 700 °C for 30 min.

The optical properties of step-oxidized ZnO thin films were studied by UV-visible spectroscopy. As discussed earlier, we observed the oxide formation of the deposited Zn films only above 400 °C.^54^ A comparison of the UV-visible spectra of ZnO films oxidized between 500 °C and 700 °C is shown in Fig. 6. Using the Tauc model and the relationship between the optical absorption and the transmittance coefficient, the optical energy bandgaps of ZnO films were successfully calculated, which were found to be 3.29 eV, 3.28 eV and 3.27 eV for oxidation at 500 °C, 600 °C and 700 °C, respectively. Overall, the estimated optical energy bandgaps of ZnO nanowires are in the UV range, which also complements our following photoluminescence (PL) results. A gradual decrease in the optical bandgap of ZnO films is clearly observed with the increase in oxidation temperature.^59^ This finding is related to a reduced optical scattering of the ZnO layers grown at higher temperatures.^60^ An increase in the particle size as well as enhanced crystalline quality is expected for ZnO films oxidized at higher temperatures. In addition, a drastic change in ZnO surface morphology is observed. All these can significantly impact and finally lead to a decrease in optical scattering and hence lower the energy band gap.^61^ These results also indicate the high crystalline quality of the ZnO nanowires.

### 3.6 Photoluminescence (PL)

**Fig. 7:**
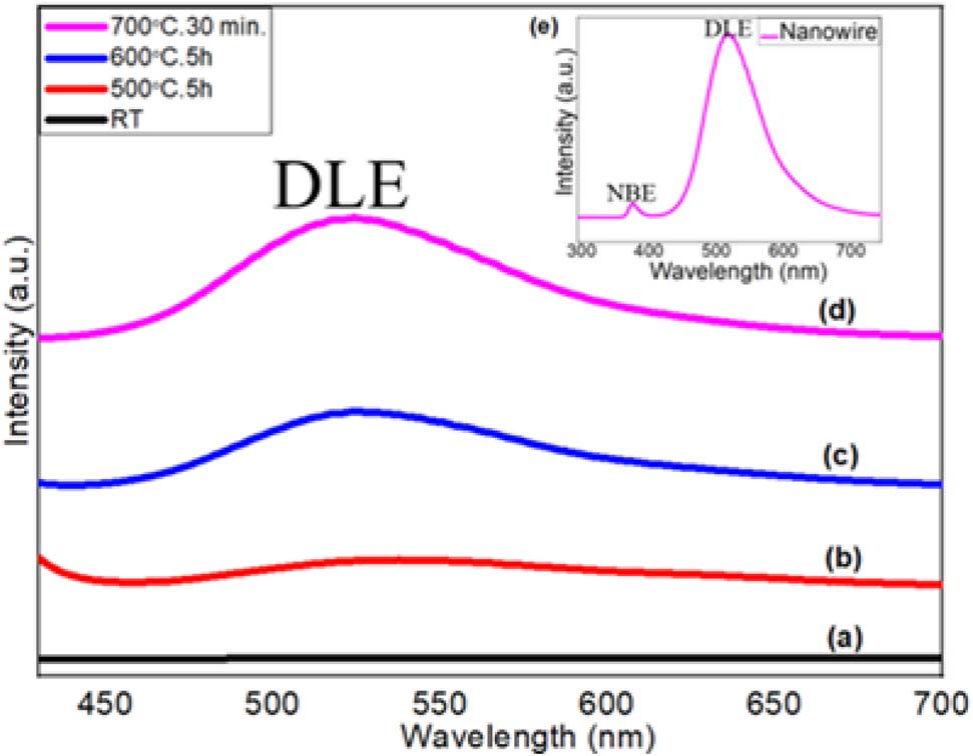
PL spectra of (a) as-deposited metallic Zn thin films at room temperature, and thermally oxidized Zn thin films at (b) 500 °C for 5 h, (c) 600 °C for 5 h, and (d and e (inset)) 700 °C for 30 min.

The photoluminescence study of ZnO films was carried out using a He–Cd laser (*λ* = 325 nm) at room temperature to observe the oxygen vacancy in terms of deep level emission (DLE) (Fig. 7). Fig. 7(a) shows the PL spectra of the as-deposited metallic Zn thin films at room temperature. No peaks are observed in the visible range (straight line), which represents the metallic characteristics of the Zn films. It suggests non-existence of any bandgap as well as band transitions within the allowed energy level. A broader peak ranging from 470 nm to 617 nm with a weaker intensity is observed at 541 nm for the ZnO layer grown at 500 °C for 5 hours (Fig. 7(b)). This visible emission generally originates from the intrinsic defect energy levels due to oxygen vacancies.^62^

Fig. 7(c) shows the PL spectra of step-oxidized ZnO thin films at 600 °C for 5 hours. The PL peak at 525 nm with a relatively strong intensity (456–602 nm) is observed here. Finally, in Fig. 7(d), the PL spectra of the ZnO nanowires grown at 700 °C for 30 minutes are presented. The PL peak appears with a much stronger intensity and is centered at 522 nm (450–595 nm). Visible broad emission peaks are mainly due to the superposition of various deep level emissions such as green, yellow and blue peaks. The deep level emission peak intensity gets stronger with the oxidation temperature due to the reduced oxygen vacancy,^63^ whereas the visible emission originates from the luminescence centers created by the interstitial Zn sites. The high intensity of DLE is due to the formation of a large number of trapped states within the bandgap range of ZnO nanowires.^64,65^ The inset shows the PL spectra of highly dense ZnO nanowires grown after step-oxidation at 700 °C for 30 minutes, where an additional weak and sharp peak of UV emission appeared at 379 nm (*E*_g_ = 3.27 eV). UV emission also known as near-band edge emission arises from the recombination of photogenerated charge carriers across the band gap. However, a very strong visible emission peak at 522 nm indicates a lower oxygen vacancy within the ZnO nanowires. These results suggest that the oxidation temperature can play a very active role in defect state density within the oxide layers.

### 3.7 X-ray photoelectron spectroscopy (XPS)

**Fig. 8:**
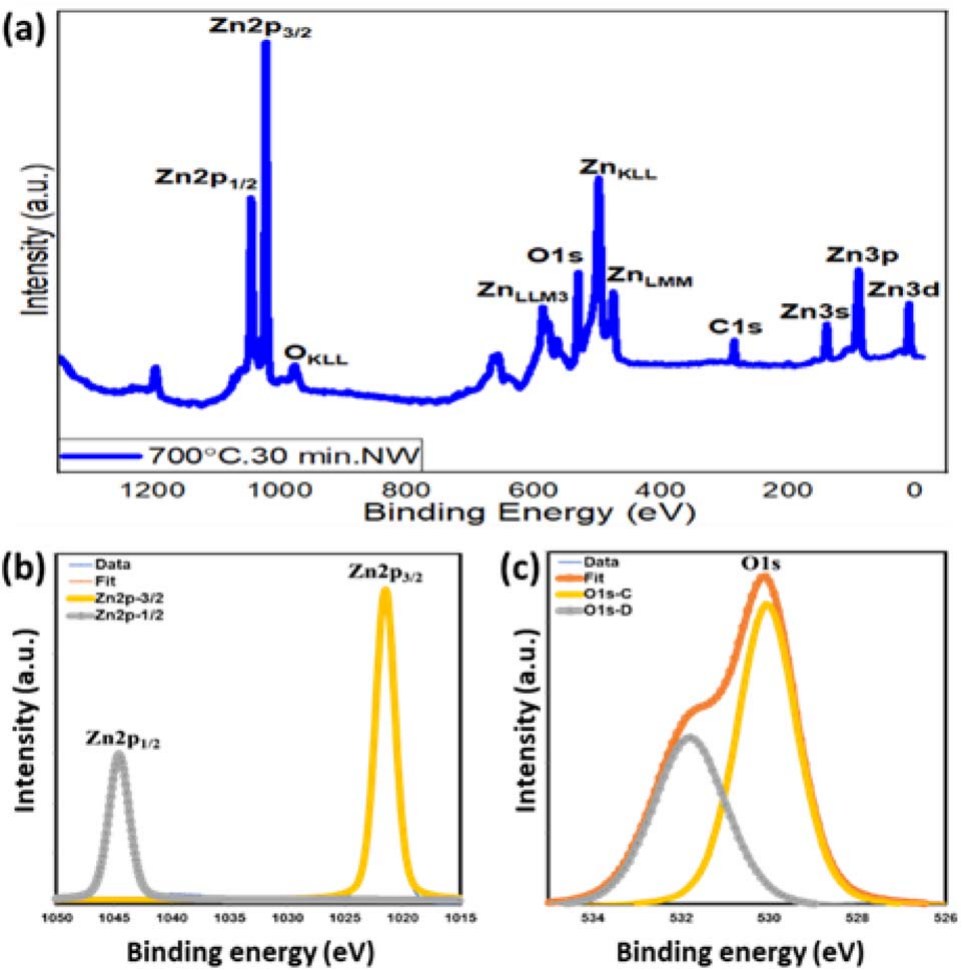
XPS binding energy spectra of ZnO nanowires grown at 700 °C for 30 min: (a) survey, (b) Zn 2p and (c) O 1s.

3.6. X-ray photoelectron spectroscopy (XPS). The surface chemical properties of the as-grown ZnO nanowires (step-oxidized at 700 °C for 30 min) were investigated using XPS technique, where Al Kα (1486.6 eV) was used as the X-ray source and the base/working pressure of the chamber was maintained below 10^−9^ mbar. Fig. 8(a) shows the XPS survey spectra of the as-grown ZnO nanowires with different elemental peaks of the ZnO nanowires. All core-level binding energy peaks are identified as Zn2p_1/2_, Zn2p_3/2_, O1s, Zn3s, Zn3p, and Zn3d and are mentioned. Fig. 8(b) shows the high-resolution scan of the Zn 2p core level spectra. A spin–orbit splitting of 23 eV is observed between Zn 2p_1/2_ and Zn 2p_3/2_ BE peaks centered at 1044.5 eV and 1021.5 eV, respectively. This finding confirms the presence of the Zn + 2 oxidation state of ZnO nanowires. Fig. 8(c) shows the high-resolution scan of O 1s, where the main BE peak centered at 530.1 eV is observed along with a shoulder peak at 532.5 eV attributed to the O_2_^−^ state present within the ZnO crystal (O 1s – C), and the surface/defect-induced states of oxygen (O 1s – D), respectively. A significantly strong surface state of O 1s also indicates the high surface-to-volume ratio of these nanowires. The presence of C 1s BE peaks at around 285.0 eV mainly due to surface contamination, which is used as the reference for this XPS study.^66^

Table 2 represents the BE peak position along with relative intensities to have a quantitative analysis of the chemical composition of the ZnO nanowires. An intensity ratio of 2/1 between Zn 2p_3/2_ and Zn 2p_1/2_ is very much in line with the theoretical values. In addition, the ZnO stoichiometry (intensity ratio between Zn 2p_3/2_ and O 1s-C) also suggests an oxygen vacancy of the ZnO crystal, complementary with the n-type nature of the ZnO films. Detailed XPS analyses of various ZnO nanostructures grown at different oxidation temperatures were also compared. Both O 1s and Zn 2p_3/2_ deconvoluted spectra appear qualitatively similar but there are some significant quantitative differences. With the increase in oxidation temperature, an enhanced O_2_ species within the ZnO film is observed. The intensity ratio of oxygen species within the ZnO crystal O 1s(C) to the zinc species Zn 2p_3/2_ relatively increases. This is a clear indication of the enrichment of oxygen content within the ZnO film grown at a higher oxidation temperature. A gradual increase in ZnO_*x*_ stoichiometry from *x* = 0.75 to 0.94 is also observed for ZnO grown at 500 °C and 700 °C, respectively.

**Table 2:** XPS BE peak data of ZnO nanowires grown at 700 °C for 30 min

No.	Element peak	BE position (eV)	Intensity (a.u.)
1	Zn 2p_3/2_	1021.5	1 240 065
2	Zn 2p_1/2_	1044.6	620 032
3	O 1s-(C)	530	162 835
4	O 1s-(D)	531.5	91 654

### 3.8 Growth mechanism of ZnO nanowires

The main aim of this work was to successfully grow extremely long ZnO nanowires of high crystalline quality and to understand their underlying growth mechanism. The 1D growth of ZnO nanowires can be explained as a combined effect of several methods, some of which are discussed as follows.

#### 3.8.1 Thermodynamics

**Fig. 9:**
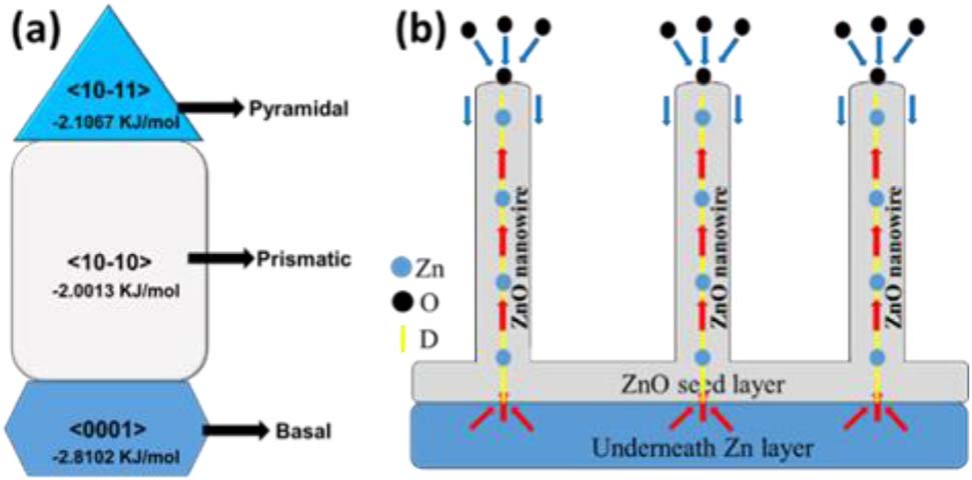
Schematic representations of (a) the ZnO crystal plane with surface free energy and (b) Zn adatom transport mechanism through defect boundaries.

Different surface free energies and growth rates associated with various crystallographic planes of ZnO significantly modulate the 1D growth of ZnO. The surface free energy of any crystal facet represents the surface thermodynamic and it prefers the growth of lower energy surfaces after sufficiently long duration of thermal oxidation at a relatively high temperature. The schematic of different crystal planes along with their free surface energies is presented in Fig. 9(a). The surface free energy of the ZnO prismatic 〈10–10〉 and pyramidal 〈10–11〉 planes was found to be significantly lower than that of the basal 〈0001〉 planes of ZnO. Therefore, nonpolar pyramidal and prismatic crystal planes of ZnO are expected to dominate over the polar basal planes during the nanowire growth under the thermodynamic equilibrium. Preferential formation of 1D ZnO nanowires with dominant nonpolar crystal planes at 700 °C is mainly due to the lower surface energy.^2^ At the same time, the lower surface energy of the Zn 〈0001〉 planes as compared to the 〈10–10〉 planes may also play a crucial role during the initial oxidation process. The lattice mismatch for the interfacial (Zn/ZnO)^10^ plane (4.8%) is significantly lower than that of the [0001] plane (21.6%), which can also influence the 1D nanowire growth along the *c*-axis of ZnO crystals.^38^ In addition, the growth rate is maximum along the *c*-axis, which could be the preferential growth direction of ZnO nanowires.

#### 3.8.2 Kinetics

The thermal mobility of Zn and O adatoms during the oxidation process can also control the 1D growth of ZnO nanowires by restricting the oxide growth only at the tip of the nanowires. The intermittent step-oxidation for a short duration usually leads to a limited/controlled surface kinetics, which significantly reduces the Zn and O adatom transport during the oxidation process. Most of the reported metal oxide nanowires are usually grown at a reduced O_2_ pressure under an Ar gas flow. The intermittent step-oxidation process may also provide a similar kind of reduced O_2_ supply during the oxide formation and eventually promote the 1D growth process.

Seed layer formation: As the O_2_ molecules come into contact with the Zn atoms at a lower oxidation temperature, a thin layer of ZnO is initially formed on top of the Zn film, which acts as a seed layer for further ZnO nanowire growth. Initial oxidation starts at the grain boundaries, *i.e.*, the surface defect sites of Zn. As the temperature increases above the melting point of Zn, the inner part of the Zn grain agglomerate together to larger grains but stress-induced continuous outer diffusion of Zn atoms through the seed layer grain boundary will continue and act as favourable nucleation sites for nanowires growth. However, beyond a certain thickness of the ZnO layer, O_2_ species cannot diffuse through it to reach the underneath Zn layer, which eventually restrict the oxidation process.

#### 3.8.3 Adatom transport

The transport mechanism of Zn and/or O adatoms across the seed layer is mainly controlled by the stress-induced mass transport through the defect boundary.^52,67^ Although both Zn and ZnO have similar hexagonal close packed (hcp) crystal structures but exhibit different lattice parameters, causing a lattice mismatch. As a result, a compressive stress was developed by the ZnO over layer to underneath Zn layer due to the volume expansion of Zn after ZnO formation (molar volumes Zn = 9.1 cm^3^, ZnO = 14.2 cm^3^). In addition, a mismatch in thermal expansion coefficients can further induce an additional compressive stress. This compressive stress causes an outer diffusion of Zn ions through the grain boundary of the ZnO layer.^6,68^ As it has been already mentioned that ZnO nanowires are preferably grown along the (0001) direction, a screw dislocation along the *c*-axis can provide the desired pathway for outer diffusion of Zn ions from the base to the tip of the ZnO nanowire. A schematic of this Zn adatom transport is depicted in a Fig. 9(b). High-resolution SEM imaging of the ZnO nanowire (Fig. 3(f)) shows significant surface roughening of the ZnO NW side wall. This can be attributed to the lateral growth of slate-like facets at the top of the NWs, starting from the core (screw dislocation) to the side wall. As molten Zn can play the role of catalytic/surfactant for a smooth ZnO layer growth, outer surface diffusion of Zn ions along the side walls of the nanowires can be excluded. Hence, we conclude that the ZnO nanowires are grown along the (0001) direction mainly due to compressive stress-driven outer diffusion of Zn ions through the screw dislocation at the center of the nanowire.

### 3.9 Gas sensing study of ZnO nanowires

**Fig. 10:**
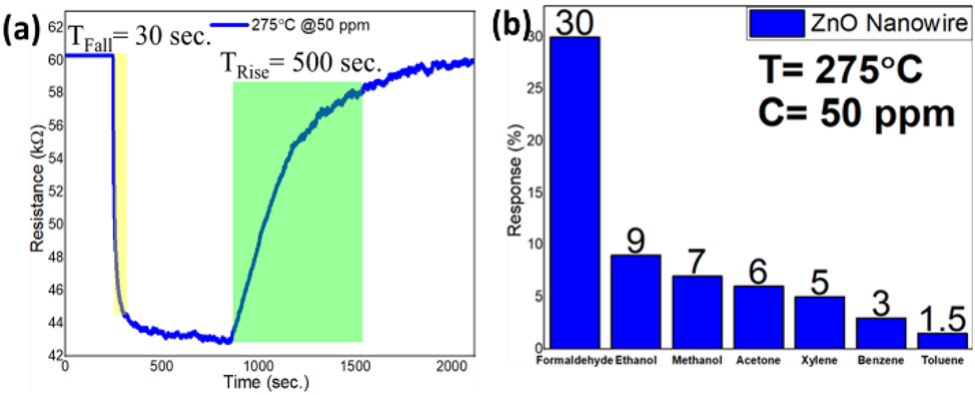
(a) Response curve and (b) selectivity chart of the ZnO nanowire-based gas senor operating at 275 °C and 50 ppm formaldehyde vapour concentration.

To emphasize on technological importance, a gas sensing study of these ZnO nanostructures was also conducted. It has been observed that the ZnO nanowire-based sensor can easily detect a very low concentration of formaldehyde vapour as low as 100 ppb. A typical response curve of formaldehyde sensing at an operating temperature of 275 °C and a formaldehyde concentration of 50 ppm is shown in Fig. 10(a). The sensitivity (*S* = Δ*R*/*R*_a_ × 100%, where Δ*R* represents the change in surface resistance during gas/vapour exposure and *R*_a_ represents the surface resistance under air ambient conditions) of the chemi-resistive sensor was found to be 30%, whereas the response and recovery times were measured to be 30 s and 500 s, respectively. Moreover, the sensor shows a very good selectivity towards formaldehyde vapour over ethanol, methanol, acetone, benzene, xylene and toluene. A response chart for selective sensing of formaldehyde vapour over other VOCs is depicted in Fig. 10(b).

## 4 Conclusions

Dense ZnO nanowires of a very high aspect ratio (length ∼60 μm and diameter ∼50 nm) are routinely achieved following a very simple thermal step-oxidation process at 600 °C for 5 hours followed by quick oxidation at 700 °C for 30 min. ZnO nanowires show a very high crystalline quality and excellent surface morphology with a strong preference of growth along the *c*-axis of the ZnO crystal. The asymmetric growth of 1D nanowires is explained in terms of surface free energies of different crystal planes, limited surface kinetics of the Zn and O adatoms within the seed oxide layer and compressive stress-driven outer diffusion of Zn ions though the defective core of the nanowire (screw dislocation). These nanowires have an extremely high surface-to-volume ratio and possess many defect states, which can be used as active materials for various applications such as UV detectors, gas sensors, light emitters, photovoltaics, photocatalysts and FE electron sources. To test these superior abilities, ZnO nanowires were used to fabricate chemo-resistive gas sensors for VOC sensing, which show an excellent sensitivity towards formaldehyde vapour and also appear with a very good formaldehyde selectivity over other VOCs. Finally, the growth methodology has also been validated through various samples.

## Data availability

The data supporting this article have already been included within the main manuscript or as part of the ESI.† However, if needed further data will be provided on request basis.

## Conflicts of interest

There are no conflicts to declare.

## Supplementary Material

RA-014-D4RA03128H-s001
