# Supplementary material for: Surface energy and stress driven growth of extremely long and high-density ZnO nanowires using a thermal step-oxidation process
Source: RSC Adv. 2024 Sep 3;14(38):28086–97. doi: 10.1039/d4ra03128h (PMC11369671; doi:10.1039/d4ra03128h)
Supplement: RA-014-D4RA03128H-s001 [file RA-014-D4RA03128H-s001.pdf]

Supplementary Information (SI) for RSC Advances.
This journal is © The Royal Society of Chemistry 2024

Surface energy and stress driven growth of extremely long and high density ZnO nanowires using thermal step-oxidation process

Sri Aurobindo Panda[a], Sumita Choudhary[a], Sushil Barala[a], Arnab Hazra[b], Suchit Kumar Jena[c], and Subhashis Gangopadhyay[a]*

Supplementary information

Statistical analysis of the as grown ZnO nanowires has been performed using FESEM imaging to validate the step-oxidation growth methodology for extremely long ZnO nanowires formation. Basically, two different statistical analyses have been adopted here. In one case, different samples are prepared during the same oxidation process (Sample S1(a-c) and S2(a-b)), whereas in other case samples are prepared through different oxidation process of similar thermal treatment (S1(a-c)-S2(a-b), S3 and S4), within a time period of six months. All samples appear with a very similar kind of ZnO nanowires surface morphology, which can successfully validate the reliability of our step-oxidation process for ZnO nanowires growth.

Table S1. Statistical analysis of the diameter of ZnO nanowires grown using similar kind of thermal step oxidation process.

Scan Date	Scan Time	Sample no.	Morphology	Diameter (nm)
09-12-2021	01:51:06 PM	S1(a)	Nanowire	50
09-12-2021	02:25:16 PM	S1(b)	Nanowire	50
09-12-2021	03:37:06 PM	S(c)	Nanowire	48
31-12-2021	02:01:35 PM	S2(a)	Nanowire	50
31-12-2021	02:25:22 PM	S2(b)	Nanowire	51
23-02-2022	12:21:17 PM	S3	Nanowire	47
21-04-2022	09:23:51 PM	S4	Nanowire	50

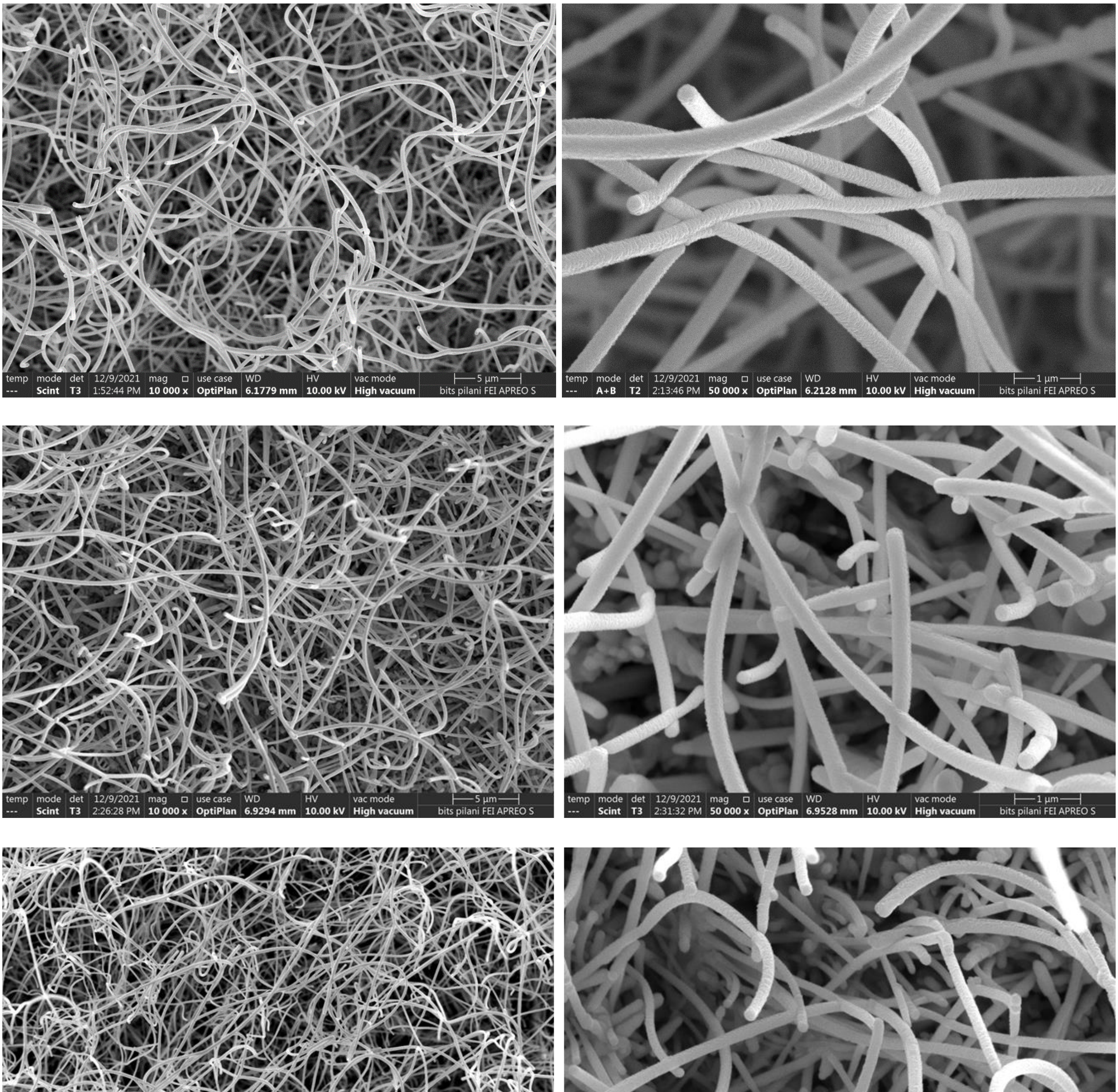

Figure SI 1(a): FESEM images of Sample S1 (a) at top, (b) in middle and (c) at the bottom.

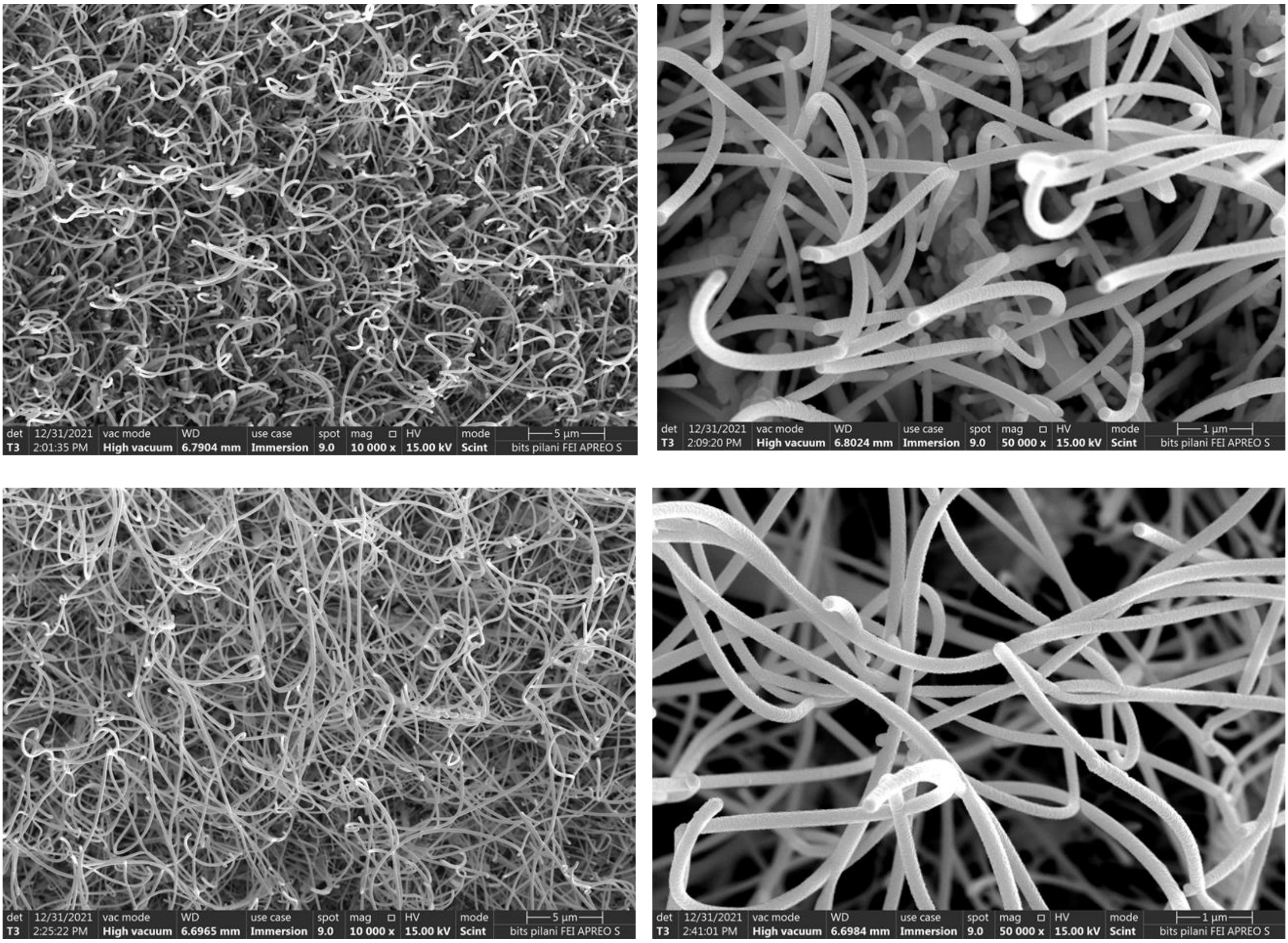

Figure SI 1(b): FESEM images of Sample S2 (a) at top, and (b) at the bottom.

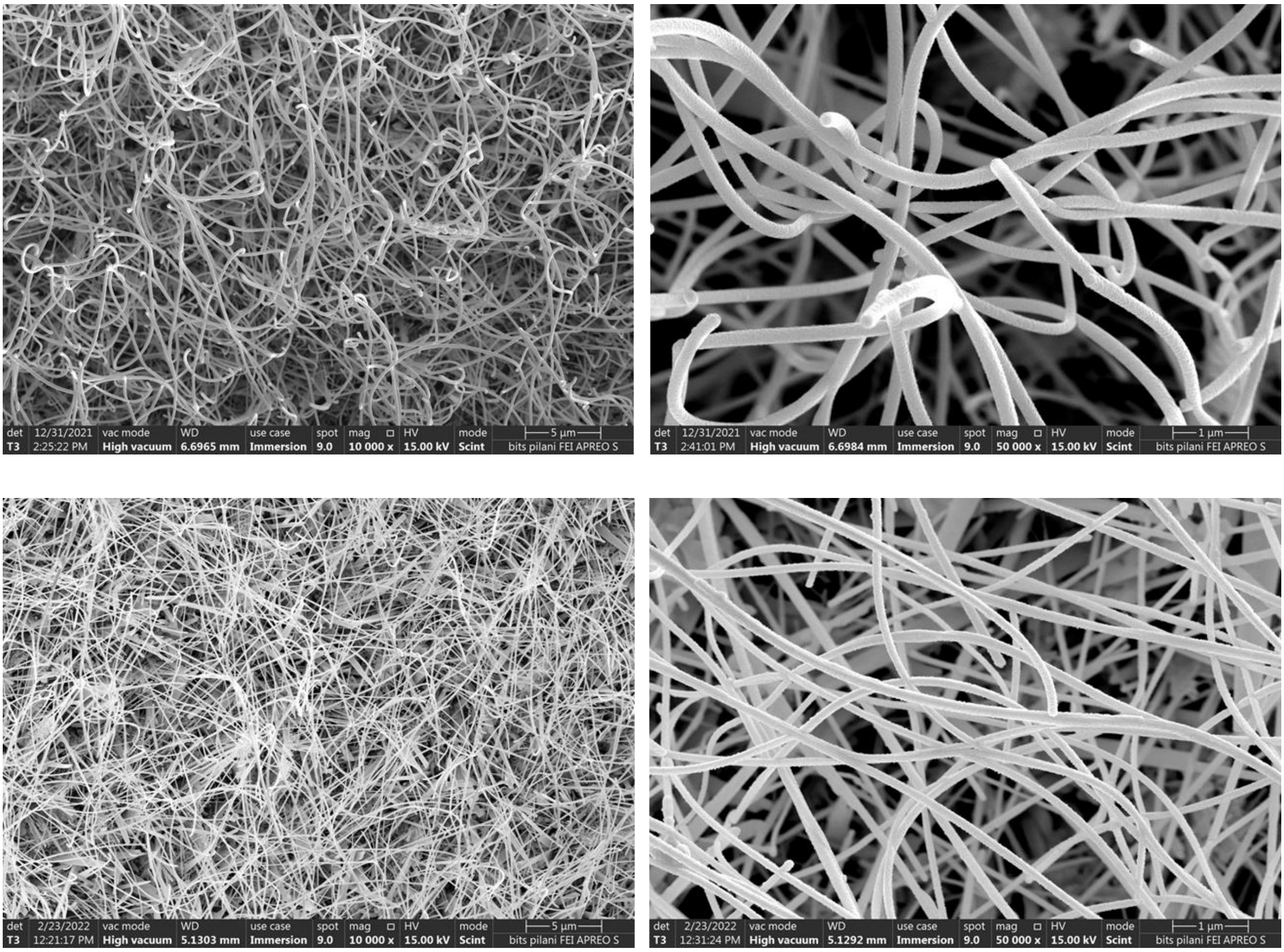

Figure SI 1(c): FESEM images of Sample S3 at top, and S4 at the bottom.
